# Norroa™ as a dsRNA biopesticide for *Varroa destructor*: insights from New Zealand research on mode of action, field efficacy, non−target effects, and social acceptance

**DOI:** 10.3389/finsc.2026.1814622

**Published:** 2026-06-09

**Authors:** Philip J. Lester, Mariana Bulgarella, Brian Manley, James D. Masucci, Rose A. McGruddy, Joana Merk, Kenneth Narva, Symon Palmer, O. Ripeka Mercier, Zoe E. Smeele

**Affiliations:** 1 School of Biological Sciences, Victoria University of Wellington, Wellington, New Zealand; 2 GreenLight Biosciences Inc., Durham, NC, United States; 3 Faculty of Medical and Health Sciences, Anaesthesiology, University of Auckland, Auckland, New Zealand; 4 Te Kawa a Māui– School of Māori Studies, Victoria University of Wellington, Wellington, New Zealand; 5 Department of Biological and Environmental Sciences, University of Helsinki, Helsinki, Finland

**Keywords:** *Apis mellifera*, calmodulin, dsRNA biopesticide, honey bee health, integrated pest management (IPM), RNA interference (RNAi), vadescana, *Varroa destructor*

## Abstract

RNA interference (RNAi) is emerging as a highly selective approach for pest management, offering species-specific control with limited non-target impacts. In apiculture, dsRNA-based biopesticides represent a novel tool for managing *Varroa destructor* (hereafter varroa), a globally distributed parasitic mite and a key driver of honey bee colony losses. Here, we review laboratory, field, and social research conducted in New Zealand that has enhanced understanding of vadescana, a dsRNA biopesticide targeting calmodulin gene expression in varroa, and its commercial formulation Norroa™. Laboratory studies using mini-hive systems demonstrate that vadescana can suppress mite reproduction, primarily through disruption of embryonic development. Varroa calmodulin and calmodulin-like gene expression was suppressed. Vadescana does not appear to affect adult mite survival. Field trials in commercial beekeeping operations in both the North and South Islands have shown that vadescana can be used to manage varroa populations and maintain low infestation levels for extended periods. The efficacy of this biopesticide in maintaining low mite populations was comparable to synthetic miticides under some conditions, but variability was observed across sites, infestation levels, and management contexts. Importantly, New Zealand-based studies indicate no or minimal impacts of vadescana on honey bee larval and adult survival, foraging behaviour, colony growth, and honey production. Bioinformatic and experimental assessments have predicted a low risk of adverse effects on non-target organisms associated with beehives. Finally, we discuss remaining challenges and future directions, including resistance management, optimisation of application strategies, incorporation into varroa integrated pest management programmes, and the need for international regulatory harmonisation. Collectively, the greatest utility of Norroa™ appears to lie in maintaining low mite populations and supporting integrated pest management programmes, rather than functioning as a stand−alone, knock−down treatment under high varroa infestation pressure.

## 1 Introduction

RNA interference (RNAi) technologies have been successfully developed as biopesticides for the control of herbivorous pests, including the Colorado potato beetle (1) and the Western corn rootworm (2). These biopesticides exploit the RNAi pathway via the delivery of sequence-specific double-stranded RNA (dsRNA) complementary to messenger RNA transcripts that encode proteins specific to, and important for, the target organism (3). dsRNA-based biopesticides are being developed for a wide variety of pathogens, parasites, and pests (4).

*Varroa destructor* (hereafter varroa) is a globally distributed mite and a leading cause of declining honey bee health and colony loss (5–7). The mite parasitises bees and is associated with hosting and transmitting a wide variety of viruses, including Deformed wing virus (5, 8, 9). The adult female mite disperses between hives and bees by clinging to adult bees, on which it feeds by piercing their exoskeleton and consuming the bees’ fat body tissue (10). To reproduce, varroa crawls from adult bees into larval brood cells, where it hides in the provisioned brood food until the cell is capped and the bee begins pupation (9). Varroa then crawls onto the pupa, pierces the soft exoskeleton, and feeds primarily on pupal bee haemolymph (11). The female mite deposits her first egg approximately three days (or 60–70 hours) after cell invasion. This first egg is male, and the foundress subsequently lays female eggs every 30 hours, which mate with their brother (5). The adult worker bee emerges approximately 12 days after pupation begins and the cell is capped. Any varroa offspring that have not completed development during this period will die. This 12-day period is thus a critically limiting factor governing the reproductive output and population growth of varroa, with average estimates suggesting as few as 1.3 viable offspring are produced during each of the adult female’s 2–3 reproductive cycles (12).

**Figure 1:**
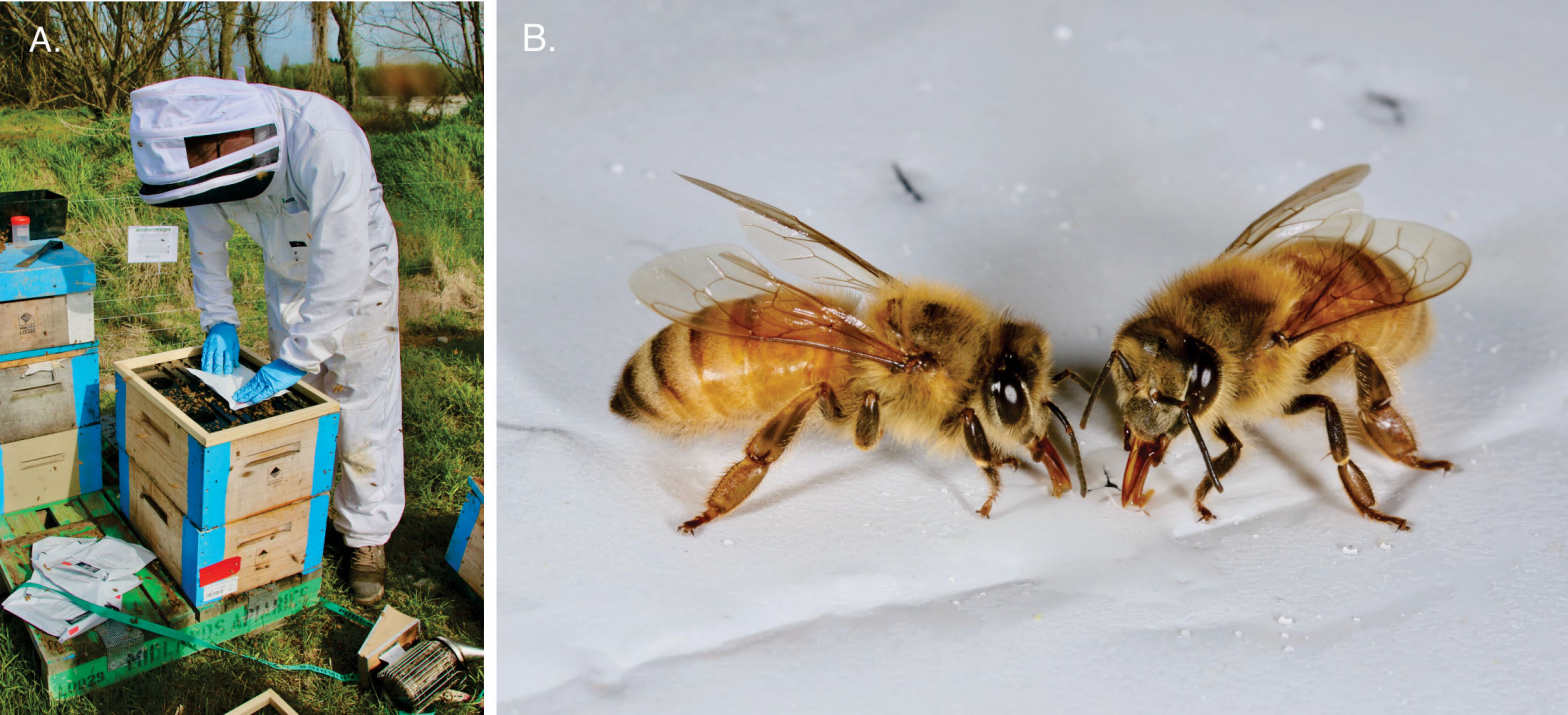
**(A)** A pouch containing 500 mL of 4 g/L vadescana in sugar water being placed into a honey bee colony in New Zealand. **(B)** Honey bees feeding on the treatment. The pouches have perforations under the label. When the label is removed the bees gain access to the product through these perforations. Photographs by Phil Lester.

Synthetic pesticides have been a key tool for beekeepers in their management of varroa. Resistance by varroa to these pesticides is becoming widespread (13–15) and has recently been associated with losses of >60% of bee colonies in the United States (16). New tools and approaches are needed for varroa management in beekeeping operations (17, 18). Researchers have explored the use of RNAi-based biotechnology as a next-generation biopesticide in apiculture to control several honey bee pests, pathogens, and parasites, including varroa. Several studies have shown that it is possible to alter gene expression in varroa after topical application or submersion of mites in dsRNA (19–22). Garbian et al. (23) demonstrated that dsRNA can be used to reduce varroa populations in laboratory colonies of bees, with recent field trials also demonstrating the considerable potential of a dsRNA biopesticide for varroa control (24, 25). GreenLight Biosciences Inc. has developed a dsRNA product, vadescana, designed to target calmodulin gene expression in varroa (24, 26). Vadescana has been used as the active ingredient in Norroa™ and is now commercially available for varroa treatment in the United States (**Figure 1**).

We have been testing dsRNA for varroa management using GreenLight Biosciences’ vadescana in New Zealand since 2022. Here, we review our laboratory and field studies assessing the use of this biopesticide, which is now commercially available in the United States as Norroa™. This review incorporates its mode of action, field efficacy in commercial beekeeping operations, studies on non-target impacts on organisms including honey bees, and the social acceptability and regulatory approval of vadescana as a dsRNA treatment for varroa management. Although RNAi−based biopesticides represent a promising alternative to conventional miticides and offer a new mode of action, resistance evolution remains a plausible risk. Experimental selection for resistance to dsRNA has been reported in other insect pests (27, 28), highlighting the need to consider resistance management proactively when developing and deploying RNAi−based products. For varroa, the potential for resistance development underscores the importance of integrating dsRNA biopesticides into broader integrated pest and resistance−management strategies.

## 2 Mode of action in varroa

Vadescana, the active ingredient in Norroa™, was designed to target calmodulin gene expression in varroa. Calmodulin binds calcium ions and enables the regulation of a wide range of enzymes, ion channels, and transcriptional regulators (29). In insects, calmodulin expression can play multiple roles, including roles in embryogenesis, development, immunology, movement, and behaviour (30–32).

We examined the mode of action of vadescana in varroa using small populations of honey bees and varroa maintained under laboratory conditions in mini-hives (33, 34). These experimental colonies are housed in modified, full-depth, five-frame corflute beekeeping nucleus (nuc) boxes. Each box was divided into two sections: a foraging area and a brood area containing a wax frame with developing bee brood. A section of wax frame is taken from a donor hive and contains bee larvae of known age, approximately 300 nurse bees, and 40–60 adult female varroa. Bees are restricted to foraging within the mini-hive. dsRNA treatments can be administered either within the hive or via a feeding station. Mite reproductive success can be estimated immediately prior to, or as adult bees emerge from pupal cells. These laboratory experiments tested concentration of 2 g/L and 8 g/L vadescana (calmodulin dsRNA) (33, 34). The concentration used in field trials (24, 35) and in the registration of the Norroa™ product was 2 or 4 g/L (36). These concentrations are within the range of other studies using dsRNA to alter varroa gene expression (19–21, 37).

**Figure 2:**
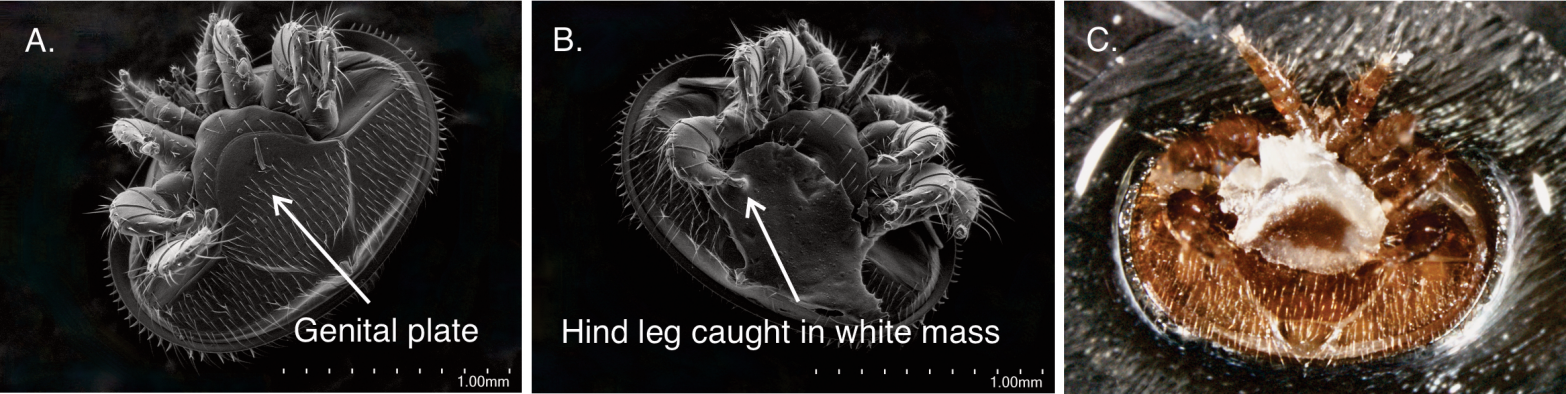
Adult female varroa. **(A)**Scanning electron micrograph (SEM) showing the ventral surface of an adult mite, including the genital plate. This plate normally folds down from a hinge near the anus, allowing oviposition of white eggs. **(B)** A mite recovered from a mini-hive treated with 8g/L vadescana. A white mass covers the genital plate and appears to originate from a malformed egg, with the right hind leg seemingly trapped within the mass. **(C)** Light microscopy image of a mite exhibiting the same white mass. SEM images **(A, B)** were taken by James Baty; the light microscopy image **(C)** was taken by Phil Lester.

Varroa mite survival in our mini-hive experiments did not appear to be affected by the dsRNA treatment (33, 34). Instead, the vast majority of foundress mites exposed to vadescana failed to produce any offspring. At vadescana concentrations of 2 g/L and 8 g/L, foundress mites produced an average of 0.13 ± 0.07 (SE) and 0.16 ± 0.07 offspring per mite, respectively. In contrast, foundresses in an inert dsRNA control and sucrose control treatments produced an average of 2.04 ± 0.25 and 2.57 ± 0.63 offspring, respectively. Some of the recovered foundress varroa from the vadescana-treated hives had acquired a white, viscous mass beneath their genital plate resembling a malformed egg mass (**Figures 2A–C**). These white masses appeared to interfere with the limbs and movement of the mites.

**Figure 3:**
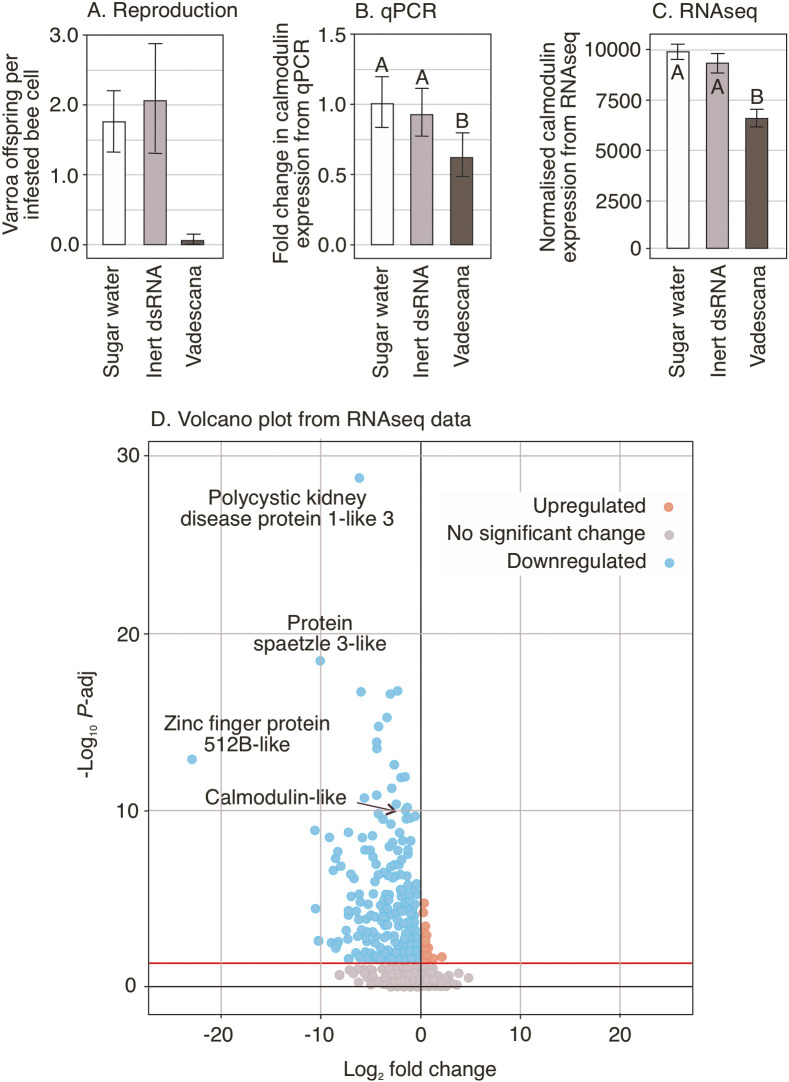
Effects of vadescana (8 g/L dsRNA) on varroa reproduction and gene expression, adapted from Smeele et al. (34). **(A)** Mean number of offspring per infested bee brood cell. Offspring include both mature and immature mites. Control treatments consisted of sugar water and an inert dsRNA control (2 g/L dsRNA targeting the soybean MPK4a gene). **(B)** Relative expression of calmodulin (LOC111247608) in varroa measured by qPCR, and **(C)** by RNAseq, 5 days after pupal bee cell capping. **(D)** A volcano plot showing up- and down-regulated genes in varroa treated with 8 g/L vadescana compared with the inert dsRNA control, five days after pupal cell capping. In B&C, significant differences (*P* < 0.05) between treatments are indicated by different letters. Error bars represent ± 1 standard error.

Smeele et al. (34) showed that two paralogous calmodulin genes were affected by vadescana treatment (**Figures 3A–D**). The expression of both varroa calmodulin (LOC111247608; **Figures 3B, C**) and a varroa calmodulin-like gene (LOC111247793) were significantly down-regulated by vadescana treatment, when measured 5 days after the bee pupal cells were capped. By the time bees were emerging from the frames (~12 days after cell capping), the expression of these genes in varroa appeared to have normalised and no significant differences in the expression of either gene were observed from RNAseq or qPCR analyses. The RNAseq data indicated that a range of genes in varroa were downregulated, many of which were probably associated with calmodulin expression. A gene ontology analysis showed that calcium ion binding and cadherin related genes that are likely to be engaged in processes associated with embryo development and cell adhesion were down-regulated in both vadescana 2 and 8 g/L treated varroa. Little is known about the function of many genes in varroa, although genes down-regulated in varroa including zinc finger proteins (**Figure 3D**) are known to have a role in the embryonic development of other insects (38). Recent studies have proposed *patch* homolog protein and AP-1 transcription factor genes involved in hedgehog and Wnt signalling pathways, respectively, to be highly important for successful varroa reproduction (20, 39). RNAi induced silencing of these genes resulted in Varroa mite infertility (20). Results from our RNA-seq dataset found expression of both these genes to be significantly reduced in vadescana 2 and 8 g/L treated varroa, suggesting their expression may be influenced by calmodulin-mediated calcium signalling (34). Significantly down-regulated processes in vadescana treated mites included those involved with segment polarity determination, post-embryonic morphogenesis, and oenocyte differentiation (‘liver-like’ cells crucial for lipid processing). These processes appear to be upregulated in mites that reproduce successfully compared to those that do not reproduce while in the brood cell (39).

## 3 Field trials and efficacy under commercial hive conditions

Field trials of dsRNA biopesticides for varroa control have demonstrated promising efficacy, but also indicate variability across sites, infestation levels, and management contexts. The first published field trials using dsRNA for varroa control were from five locations in the United States (24). These results showed that treatment with 4 g/L vadescana in the spring could maintain mite numbers lower than a 3 varroa/100 bee treatment threshold for 18 weeks, and varroa densities were significantly lower than in hives treated with either amitraz or without any mite control (24). A subsequent Italian study used a mixture of dsRNA targeting varroa acetyl-CoA carboxylase, ­ Na+/K+ ATPase, and endochitinase genes. These Italian field trials showed reduced mite infestation rates 37 days after treatment, as estimated from varroa post/pre-infestation ratios (25).

**Figure 4:**
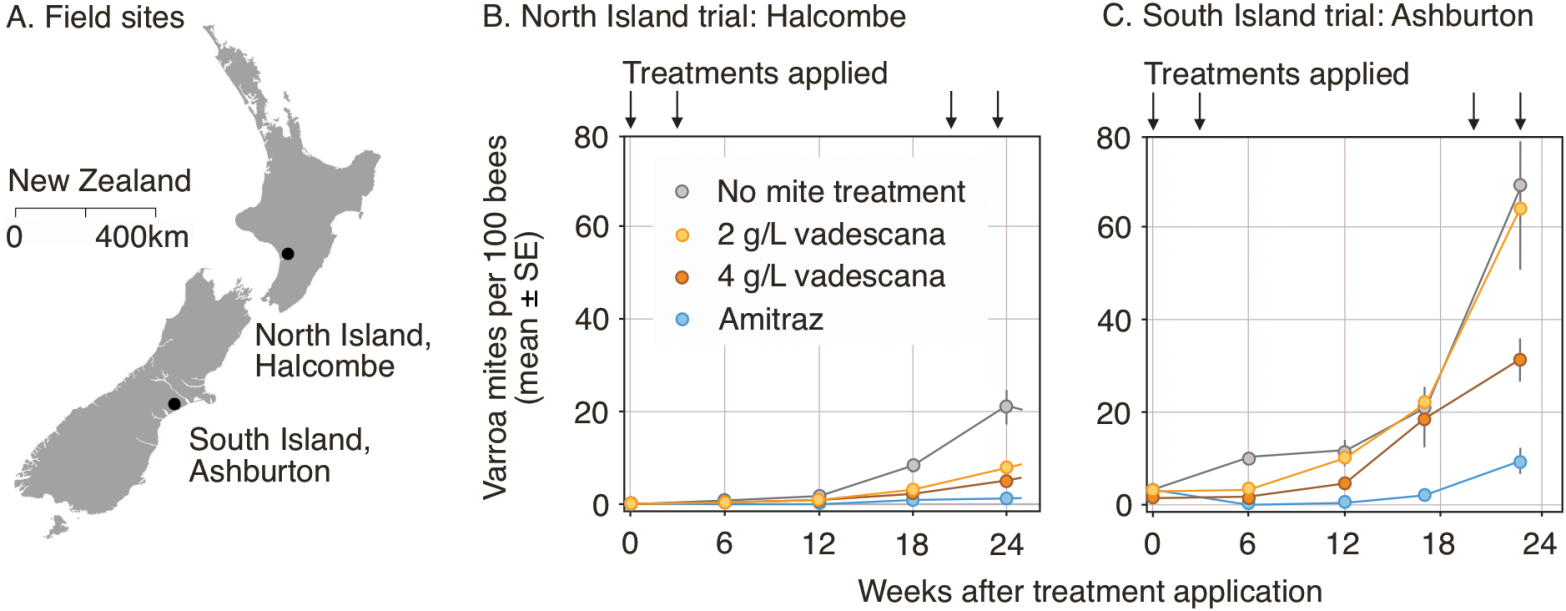
**(A)** Field sites for New Zealand field trials. Trials at each site were initiated with 40 honey bee colonies in each of the four treatments. **(B, C)** Varroa densities from New Zealand field trials using vadescana (2 g/L vadescana and 4 g/L), compared to the use of synthetic pesticide (amitraz) and a sucrose solution (untreated) control treatment. Values are the means ± 1 standard error, initially with 40 hives in each treatment. Data are from McGruddy et al. (35).

Field trials with vadescana in New Zealand used two vadescana concentrations of 2 and 4 g/L (35). Two trial sites were used, one in the North Island and one in the South Island (**Figure 4A**), with mite populations in hives followed for up to 44 weeks. Twelve weeks after trial initiation, both vadescana treatments significantly reduced mite populations compared to the untreated controls, with densities comparable to the synthetic miticide treatment (**Figures 4B, C**). After this 12-week period, at one of the two study sites mite populations were maintained at relatively low densities (**Figure 4B**), while mite populations attained high densities exceeding 20 mites per 100 bees after 18 weeks at the South Island site (**Figure 4C**). These contrasting outcomes strongly suggest that vadescana efficacy is sensitive to initial colony size and infestation intensity, although the relative importance of these and other factors (e.g., brood availability, seasonal timing) was not experimentally disentangled in these trials.

Collectively, these results indicate that vadescana can contribute to varroa management, but also that its efficacy is strongly contingent on infestation intensity and colony context, and that application rates may need to be adjusted according to hive size. In contrast to knock−down miticides, vadescana appears best suited to maintaining low mite populations rather than suppressing high, rapidly growing infestations (35). This interpretation is supported by field trials in Florida, where vadescana in Norroa™ did not significantly suppress high mite populations but was effective when initial infestation levels were below damaging thresholds (40). Together with the New Zealand trials, these results suggest that the current formulation is most appropriately deployed as a preventative or maintenance treatment rather than as a stand−alone curative intervention (40). Additional research is therefore needed to define operational thresholds for effective use, including infestation intensity, colony size, and optimal timing of application under different seasonal and management conditions.

McGruddy et al. (35) also measured honey production at the two sites of this field trial. At the North Island site there was no significant difference between dsRNA or other treatments in the honey crop harvested 18 weeks after the initiation of the trial. The South Island site showed that honey production in both the 2 g/L and 4 g/L vadescana treatments was significantly higher than that from colonies without mite control (35). As with mite abundance, honey yield responses were influenced by site−specific conditions and background colony performance, limiting the extent to which productivity outcomes can be generalised across trials. The site−specific responses, however, suggest that benefits to honey production are most likely to arise where effective varroa control alleviates constraints on colony performance.

## 4 Honey bee survival and foraging after vadescana treatment

Many existing synthetic pesticide treatments for varroa mites affect bee health. Gashout et al. (41), for example, found that honey bee foraging was significantly reduced by pyrethroid, organophosphate and organic acid treatments for varroa. Amitraz is another globally used pesticide for varroa management, which can reduce larval bee survival (42). Hence, we have examined the influence of vadescana on the survival of larval and adult bees, and the adult foraging behaviour. We also report on the survival and growth of commercial colonies treated with vadescana from our field trials.

McGruddy et al. (33) exposed bee larvae to varroa at both a 2 g/L and an 8 g/L vadescana treatment, 2–3 days prior to the bees capping the cells in laboratory mini-hives. The cells were uncapped 12 days later, immediately before the bees would likely have emerged. While varroa reproduction was substantially reduced, approximately 95% of bee pupae survived and appeared unaffected by the biopesticide treatment. The bees that emerged from these experimental treatments in the laboratory showed no evidence of any developmental irregularities (33). We note that these experiments were designed primarily to test the dsRNA effects on varroa and only examined mature bee larvae. Future work could examine earlier instar bee larvae, which were not the focus of these experiments.

**Figure 5:**
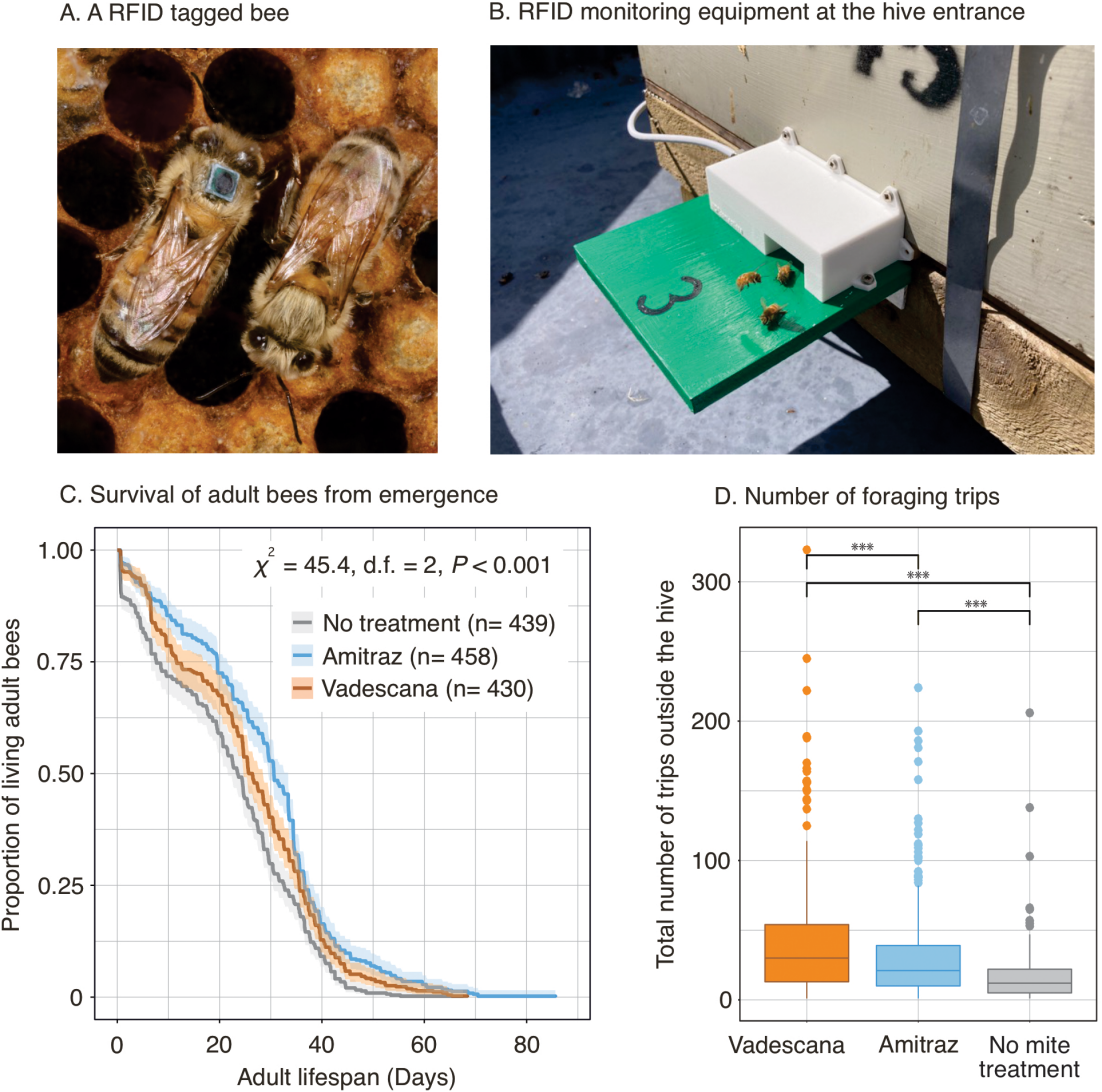
Experiments examining how varroa control treatments (including dsRNA) influences worker bee longevity and foraging. Honey bees were reared as juveniles in hives treated with vadescana (4 g/L dsRNA), amitraz, or in colonies with no mite management treatment. **(A, B)** As worker bees emerged from pupal cells, each was fitted with an RFID (Radio Frequency Identification) tag. An additional vadescana treatment was added to the hive after bee emergence and tagging. **(C)** A Kaplan–Meier plot showing significant differences in the mean survival of bees among treatments. The shaded area represents 95% confidence intervals. **(D)** The total number of foraging trips per adult bee over its lifetime. Boxes extend from the first to the third quartile, whiskers extend to 1.5 times the interquartile range, and values outside this range are plotted as individual points. Significance brackets and *** indicate groups that were significantly different at *P* ≤ 0.001. Data are from Merk et al. (43). Photographs by Phil Lester.

The foraging and survival of adult honey bees were investigated using radio frequency identification (RFID) tags, which enable individual bee survival and foraging to be monitored over their entire lifespan (43). The bees were reared in field colonies under different varroa treatments including with vadescana treatments, tagged on the same day as they emerged from the cells, and then immediately placed back into the donor hives. Results from this study indicated that varroa substantially influences adult bee survival (**Figures 5A–C**). Adult bees from colonies without varroa control had the shortest average lifespan of just 21.8 days (95% CI = 20.5-23.2). Those from amitraz-treated hives had the longest average lifespan 28.7 days (95% CI = 27.4-30.1), with bees from vadescana-treated colonies intermediate at 25.4 days (95% CI = 24.0-26.8) (43). Significant effects of varroa control were also observed regarding foraging behaviour (**Figure 5D**). Vadescana treated bees on average started foraging 8.5 days after emerging as adults, which was 1.9 days sooner than bees from amitraz-treated colonies and 4.1 days before bees from colonies without any varroa control. Bees from vadescana-treated colonies made more foraging trips and had the shortest foraging trip length. Their foraging behaviour may have contributed to the observed slightly shorter lifespan of vadescana-treated honey bees compared to amitraz-treated honey bees (43).

The results from laboratory trials examining juvenile bee survival and RFID studies appear to correspond with honey bee colony growth patterns from the large-scale field trials. These field experiments indicated that 4 g/L vadescana-treated colonies increased in colony size to significantly higher populations than in honey bee colonies without varroa control, and then those in colonies where mites were successfully controlled with amitraz, for at least 12 weeks post-treatment (35). These results showing little influence of vadescana on juvenile and adult bee survival and foraging were expected given the lack of dsRNA target sequence homology between the varroa calmodulin gene and with the honey bee genome (44). We note other reports that non-specific dsRNA sequences can elicit changes in gene expression profiles in honey bees, with downregulation of genes associated with immunity or responses to stress (45, 46). Future work could examine potential non-specific effects of dsRNA on honey bee gene expression and fitness under varying biological and environmental conditions.

## 5 Potential non-target effects on other arthropods

The genomes of other arthropods have been examined bioinformatically for potential non-target effects of the vadescana dsRNA sequence. Bulgarella et al. (44) examined the genomes of 39 invertebrate species, with the vadescana 402-bp sequence compared against each genome. The total number of siRNA hits was calculated to estimate how many of those matches could efficiently activate the siRNA machinery.

We found the highest number of genomic matches (260) were to the genome of the parasitic mite related to varroa, *V. jacobsoni*. Six other species had predicted siRNA hits of 21 nt length: the monarch butterfly (*Danaus plexippus*), the fruit fly (*Drosophila melanogaster*), the European earwig (*Forficula auricularia)*, and three species of mites (*Stratiolaelaps scimitus*, and two *Tetranychus* spp.) (44). We note that having predicted siRNA hits does not confirm any impact on gene expression. For example, Krishnan et al. (47) also observed a 21-nt hit of the vadescana sequence to the monarch butterfly genome. However, in feeding experiments they observed no significant effect of the dsRNA on monarch larval mortality, larval or pupal development, pupal weights, or adult eclosion rates. Their experiments involved feeding caterpillars with leaves sprayed with a dsRNA concentration of 21 g/L, which is five-fold higher concentrations than that used in vadescana. They concluded that monarch mRNA may be refractory to silencing by dsRNA or that the butterfly might degrade the dsRNA to a concentration that is insufficient to silence mRNA signalling (47). Many insect species appear not to be affected by orally ingested dsRNA, due to a range of mechanisms including by being deficient in core RNAi molecular machinery (48).

We note that while such *in silico* approaches are valuable for identifying potential non−target hazards, consideration of realistic exposure scenarios is essential for determining the likelihood and magnitude of non−target effects under field conditions. In a practical sense, it seems unlikely that monarch butterflies or most other arthropods, including the European earwigs or *Tetranychus* mites, would come into contact with vadescana. Beekeepers place the dsRNA inside hive boxes, within the colony, where bees aggressively defend the beehive in order to protect their honey crop.

Some insects, however, can co-reside within honey bee colonies including the greater wax moth (*Galleria mellonella*). Bulgarella et al. (44) used an experimental design that represented an extreme and unlikely worst-case scenario in which consecutive wax moth generations were continuously exposed and fed vadescana. No significant effects were observed in wax moth calmodulin gene expression, reproduction, survival, or adult F2 wing length between vadescana-fed and control groups. The body weight of the male F2 generation was slightly but statistically significantly lower in wax moths exposed to the highest vadescana dose, with no such effect observed in female moths. We concluded that this significant effect on the male F2 generation was likely driven by high sample numbers and low levels of variation (44). These experiments with wax moth represent conservative, worst−case exposure scenarios for selected non−target species, but we acknowledge they do not encompass the full diversity of arthropods that may be associated with beehives or surrounding environments.

Multi−year trials of another dsRNA biopesticide, Calantha™, targeting the Colorado potato beetle detected no adverse effects on non−target arthropods including on other beetles and beneficial predators and parasitoids, in contrast to conventional insecticides (49). dsRNA biopesticides have been shown to have high specificity in other pest systems (50, 51). The delivery of vadescana as a dsRNA biopesticide directly inside the beehive, combined with bee defence effecting the exclusion of many other arthropods, makes this an especially highly-targeted pest management treatment. Nevertheless, it is possible that other arthropods might be affected by vadescana or other dsRNA biopesticides, with concern that genes involved in developmental and embryonic pathways of honey bees share sequence similarity with some proposed biopesticidal dsRNAs (52). De Neef et al. (53) recommend a three-pronged risk assessment approach for examining non-target effects of dsRNA biopesticides: (i) a phylogenetic analysis of gene orthologs that defines the taxonomic scope of sequence similarity, (ii) wide searches of large databases to identify potential unexpected similarity in distantly related species, and finally (iii) full transcriptome analyses in non-target organism species of particular concern for a thorough understanding of all potential hazards. We also recommend experiments involving exposing or feeding the dsRNA to all the life-stages of non-target organisms (44, 47), but acknowledge such that experiments are costly, and may be unfeasible for some arthropods that are difficult to artificially culture. As with all emerging biopesticide technologies, uncertainty may remain regarding chronic effects, although the rapid degradation of dsRNA and limited exposure pathways reduce the likelihood of long-term effects. Structured environmental risk assessments that consider both hazard and plausible exposure scenarios across ecological contexts are necessary for thorough evaluation.

dsRNA biopesticides should rapidly degrade after application, even within 2 days (54, 55). However, dsRNA stability can be context− and formulation−dependent, with slower degradation reported in protected matrices (55), highlighting the importance of confirming dissipation rates under relevant use and exposure scenarios. It is possible that humans or other vertebrates might consume dsRNA residues in honey or beehive products including propolis. It appears highly improbable that systemic absorption of intact dsRNA would occur in humans or other vertebrate following dietary exposure of RNAi-based biopesticides. There are multiple biological barriers for dsRNA absorption at the gastrointestinal, bloodstream, and cellular levels in mammals, which typically result in ingested RNAs undergoing rapid metabolism and clearance (56, 57).

## 6 Government registration and acceptance by beekeepers and consumers

There is a strong global trend away from broad-spectrum and persistent pesticides towards more selective pest-control products based on sound biological understanding, a shift that is also being experienced in New Zealand (58). Countries regulate dsRNA−based biopesticides in markedly different ways, a phenomenon referred to as “regulatory divergence”, which has hindered their broader adoption (59). For example, the European Union currently regulates dsRNA biopesticides as chemical pesticides through the European Food Safety Authority, whereas the United States assesses them on a case−by−case basis with specific provisions for biochemical pesticides, and several countries in Asia and Australia regulate them under existing agricultural chemical frameworks (59). There is a clear need for greater global regulatory cohesion and internationally harmonised regulations and risk assessments. In the United States, the Environmental Protection Agency (US EPA) has registered Norroa™ as a dsRNA biopesticide to target varroa, concluding that it meets regulatory and safety standards under the US Federal Insecticide, Fungicide, and Rodenticide Act (FIFRA), the Federal Food, Drug, and Cosmetic Act (FFDCA), and the Endangered Species Act (ESA). The US EPA concluded that “*there is a reasonable expectation of no discernible effects to occur to any NTOs* [non-target organisms] *not inhabiting honey bee hives, and this finding would apply to all TES* [threatened and endangered species] *as these species are not reasonably expected to inhabit honey bee hives*” (36).

In New Zealand, pesticides and biopesticides are regulated under the Hazardous Substances and New Organisms (HSNO) Act 1996 and the Agricultural Compounds and Veterinary Medicines Act 1997 (ACVM Act). Like many countries, New Zealand has yet to register the use of any dsRNA biopesticide. However, a governmental ruling determined that cells and organisms treated with dsRNA should not be considered genetically modified organisms, a position that was subsequently affirmed following a legal challenge (60, 61). dsRNA biopesticides have a novel mode of action that is likely to be questioned by users and consumers wherever their use is proposed.

We surveyed beekeepers from six beekeeping clubs across New Zealand regarding their attitudes towards, and likelihood of using, dsRNA as a biopesticide for varroa control (62). This survey was conducted through six beekeeper clubs and included both hobbyist and commercial beekeepers, with responses from 175 participants. The majority (93%) of respondents reported either no concerns about the use of RNAi for varroa control or indicated that they would at least consider using it in their beehives. dsRNA was generally perceived as a non-toxic alternative to existing pesticide options, although concern was expressed by some. These concerns included potential long-term effects of dsRNA treatment on bee health and possible impacts on non-target species associated with beehives. Approximately 10% of the New Zealand beekeepers who we surveyed indicated reservations or uncertainty about consuming honey products containing dsRNA residues. Some respondents also confused RNAi with genetic modification. Despite being provided with explanatory information, 10% of surveyed beekeepers believed dsRNA involved genetic modification, and a further 26% were unsure whether this was the case (62).

Confusion between genetic modification and RNAi approaches is also evident within the general public, with limited understanding of the RNAi mode of action (63). The distinctions have been elucidated in social studies with ‘primers’ or explanatory material (62, 64–66). The development of effective two-way communication requires interdisciplinary research teams and increased time and commitment from both researchers and participants. In the context of broader pest control in New Zealand, Mercier et al. (66) found that individuals who are familiar with RNAi technology are typically highly supportive of its use. They concluded that these participants support the use of genetic pest control technologies, including dsRNA, and endorse government investment in their research, provided that risks are appropriately mitigated and development proceeds through an open process that accounts for diverse social and cultural perspectives, including concerns of Indigenous communities (66). Trust in science and scientists accompanied by educational resources that acknowledge multiple perspectives and interests in technology will support meaningful engagement (67, 68). Efficacious relationships with Indigenous peoples or the traditional land and biodiversity stewards will be necessary to shape legislative processes (65, 69).

Beekeeper and broader survey participant attitudes in New Zealand are largely reflective of attitudes towards RNAi technologies in other globally distributed primary industries. For example, a recent survey assessed United States consumers’ perceptions and attitudes towards RNAi technology in the beef sector (70). Approximately 67–69% of respondents indicated that the use of RNAi to improve animal health or protect cattle from disease was highly desirable. However, nearly two-thirds of respondents expressed concern that any potential side effects from consuming RNAi-derived products in beef are largely unknown. Women and older individuals were less accepting of RNAi technology in the beef industry, whereas respondents with a college degree were more receptive to its use (70). Very similar patterns were observed in a survey examining attitudes towards the use of RNAi in strawberry production in Europe (71).

The assessment and registration of the Norroa^TM^ by the US government is groundbreaking for dsRNA biopesticides. However, evidence from studies on varroa, beef, strawberries, and broader pest control indicates that considerable work remains to achieve public acceptance of dsRNA-based technologies. Tardin-Coelho et al. (72) suggest that, to obtain social licence for the use of RNAi-based biopesticides, practitioners and regulators should focus on building trust, improving knowledge and awareness of the technology, employing effective communication and education strategies, and ensuring early engagement and dialogue with stakeholders. The establishment of such social licence will, in turn, influence governmental and legislative processes for the registration of dsRNA biopesticides, including those developed for varroa control.

## 6 Conclusions and future work

The ideal pest-control method is highly selective, targeting specific pests without harming beneficial arthropods or vertebrates, breaks down quickly, is inexpensive, effective at low doses, safe to handle, and targets pests without fostering resistance when used appropriately. dsRNA biopesticides possess many of these attributes and have therefore been described as a potential “perfect pesticide” in principle (73). For pests such as varroa, we have found dsRNA can be effective, highly specific, with minimal impacts on non-target species, including honey bees. Norroa^TM^ is now a commercially available option for mite management in the United States. Cost and scalability have previously limited the adoption of dsRNA biopesticides. GreenLight Biosciences, however, have targeted a production rate of 20 tonnes per year at a cost of less than US$1 per gram (73). Importantly, evidence from laboratory and field studies indicates that dsRNA biopesticides such as vadescana are unlikely to function as stand−alone, knock−down treatments under high varroa infestation pressure. Instead, their greatest utility appears to lie in maintaining low mite populations, supporting integrated pest management programmes, and potentially delaying the evolution of resistance to conventional chemical miticides (18).

Challenges remain and will guide future work on dsRNA biopesticides. Additional research may be useful to further refine dosage, application frequency, and deployment strategies under different colony sizes, infestation intensities, and seasonal conditions. Resistance development to dsRNA biopesticides is possible within a relatively small number of insect generations (27, 28). The implementation of insect resistance management strategies will be essential for their long−term effectiveness in varroa control, including rotation, the integration with other control methods, and careful consideration of deployment intensity (74). The identification of additional gene targets, including the potential use of multiplexed dsRNA formulations, could further improve efficacy and mitigate resistance risk (75). Further work is also required to define how dsRNA biopesticides can be optimally integrated with existing chemical, cultural, and mechanical control tools within varroa integrated pest management programmes. Although available evidence indicates low risk to non−target organisms, continued risk assessment under a wider range of ecological and use scenarios will be important as dsRNA biopesticides move towards broader adoption. Finally, because dsRNA biopesticides are novel and unfamiliar to many users and consumers, continued attention to regulatory clarity, risk assessment, and transparent engagement will be necessary to maintain confidence and social licence. Greater international regulatory alignment and clear guidance on risk assessment will be critical to support wider adoption of dsRNA biopesticides across apicultural systems globally.
